# Dynamic multimodal brain function monitoring enables quantitative severity grading and prognostic prediction in pediatric neurocritical care

**DOI:** 10.1016/j.jped.2026.101601

**Published:** 2026-09-05

**Authors:** Yanmei Wang, Haili Wang, Minglei Li, Xianli Shao, Huafeng Zhao

**Affiliations:** Department of Pediatric Internal Medicine One, Weifang People's Hospital, Weifang City, Shandong Province, China

**Keywords:** Multimodal brain function monitoring, Pediatric neurocritical care, Perturbation factor, Severity grading, Prognostic prediction

## Abstract

**Objective:**

Multimodal Brain Function Monitoring (MBFM) enables simultaneous assessment of cerebral blood flow, intracranial pressure (ICP), and brain tissue oxygenation, providing dynamic evaluation for pediatric neurocritical care. This study evaluated MBFM-derived parameters, including perturbation factor (PF), edema factor (EF), ICP, and regional cerebral oxygen saturation (rSO₂), for disease severity stratification and prognostic prediction.

**Methods:**

We prospectively enrolled 120 pediatric patients (6 months–12 years) in the PICU of the Children’s Hospital, the Weifang People's Hospital (January 2022– November 2025). Continuous MBFM monitoring was performed for 72 h. Patients were stratified into mild, moderate, and severe groups using K-means and hierarchical clustering. Key predictive thresholds were identified via CART decision tree. Predictive performance for adverse outcomes, including Glasgow Outcome Scale scores, complications, and survival, was assessed by ROC analysis, logistic regression, Kaplan-Meier survival analysis, and decision curve analysis.

**Results:**

PF, ICP, EF, and rSO₂ significantly differed among severity groups. PF predicted adverse outcomes with an AUC 0.86 (cutoff 0.34), while the combined model (PF + EF + ICP + rSO₂) improved the AUC to 0.91. Patients with PF > 0.34 and severe disease showed reduced 6-month survival (Log-rank χ² = 7.76, P = 0.005).

**Conclusions:**

MBFM provides a quantitative framework for grading pediatric neurocritical illness. PF is a core predictor, and integrating multimodal metrics enhances prognostic accuracy, guiding PICU interventions and outcome prediction.

## Introduction

Pediatric neurological critical illnesses remain a major cause of mortality and long-term disability worldwide. Traumatic brain injury, viral encephalitis, and toxic-metabolic encephalopathy are among the leading causes of pediatric intensive care units (PICUs) admissions and are associated with poor outcomes [1,2]. Despite advances in neuroimaging, neurocritical care, and biomarker discovery, mechanisms of secondary brain injury remain incompletely understood, and effective physiological targets for intervention are limited [3,4]. Current management relies mainly on conventional parameters such as intracranial pressure (ICP) and perfusion pressure (CPP) [5,6]. However, these static, unidimensional parameters are insufficient to capture the dynamic and heterogeneous nature of pediatric brain injury, particularly in the context of development changes in cerebrovascular autoregulation, cranial metabolism, and age dependent intracranial physiology [[7], [8], [9]]. Consequently, there is growing interest in multimodal monitoring approaches that integrate multiple physiological signals to better characterize brain state and guide individualized therapy.

The pediatric brain undergoes continuous physiological maturation and should not be considered a homogeneous entity across age groups [8]. Cerebrovascular autoregulation, cerebral blood flow, and cerebral metabolic demand exhibit pronounced age-development changes, leading to substantial variation in normal physiological ranges, leading to substantial variation in normal physiological ranges and responses to acute brain injury [10]. Consequently, applying uniform physiological thresholds across pediatric may obscure clinically relevant biological heterogeneity and reduce the generalizability and applicability of neuromonitoring-based phenotyping algorithms [9]. Although multimodal neuromonitoring has gained increasing attention in adult neurocritical care, its application in children remains limited by small sample sizes, heterogeneous monitoring strategies, and the lack of standardized approaches for integrating physiological signals into clinical decision-making [11,12].

In this study, we hypothesized that dynamic multimodal physiological signatures derived from synchronized monitoring of ICP, cerebral oxygenation, and derived functional reactivity indices can identify distinct cerebral states and predict neurological outcomes in critical illness children. We therefore combined prospective multimodal monitoring with machine-learning-based phenotyping to establish a quantitative framework for pediatric neurocritical illness stratification and prognostic assessment.

## Material and methods

### Study population and data source

This prospective observational study included 120 pediatric patients (6 months-12 years) with acute neurological critical illness admitted to the Pediatric Intensive Care Unit (PICU) of the Weifang People's Hospital between January 2022 and November 2025. This research was approved by the institution ethics committee. Consecutive requiring ICP monitoring were enrolled. Inclusion required complete synchronized recordings of ICP, mean arterial pressure (MAP), regional cerebral oxygen saturation (rSO_2_), perturbation factor (PF), and electroencephalographic features (EF). Continuous multimodal monitoring was performed for 72-hour after clinical stabilization to capture early secondary brain injury evolution, including intracranial hypertension, cerebral edema, and impaired cerebrovascular regulation(6).

To account for developmental heterogeneity, patients were stratified into three age groups: infants/toddlers (6 months-3 years), young children (> 3–6 years), and school-age children (> 6–12 years). The consistency of PF thresholds and clustering-derived phenotypes across age groups was assessed using stratified ROC analysis and cluster reproducibility analysis.

### Multimodal brain function monitoring procedures

Multimodal brain function monitoring (MBFM) integrated synchronized physiological signal streams: (i) ICP reflecting global intracranial dynamics; (ii) rSO_2_ reflecting cerebral oxygen delivery-utilization balance; and (iii) MAP representing systemic perfusion driving cerebral blood flow.

ICP was continuously measured using an intraparenchymal microsensor (Codman ICP MicroSensor, Codman & Shurtleff, USA). Cerebral oxygenation was monitored using near-infrared spectroscopy (INVOS 5100C, Medtronic, USA), and MAP was obtained through continuous arterial blood pressure monitoring.

All signals were digitized at 100 Hz and recorded using ICM + software (University of Cambridge, UK). Data preprocessing included artifact removal and exclusion of segments with > 5% signal dropout.

### Physiological relationale of derived indices (PF and EF)

PF was defined as a dynamic index reflecting cerebrovascular pressure-flow-oxygenation coupling. It was calculated from synchronized fluctuations in ICP, MAP, and rSO_2_ using the root mean square (RMS) of detrended coherence deviations within a 10-second sliding window, normalized to a stable 5-minute baseline period: PF = RMS[ΔCoh(t)/Coh_baseline], where ΔCoh(t) represents the deviation of the instantaneous coherence among ICP, MAP, and rSO_2_ from the baseline coherence.

Higher PF values indicate greater disruption of integrated cerebrovascular reactivity and impaired autoregulatory dysfunction [13]. The concept is consistent with previous studies demonstrating that pressure reactivity index (PRx) can serve as surrogate markers of cerebrovascular autoregulation, with emerging pediatric evidence supporting their feasibility in neurocritical care [14].

EF was derived from non-invasive cerebral bioimpedance monitoring and represents impedance variation associated with changes in tissue water content and intracranial compliance [15]. The system uses bilateral forehead electrodes to record cerebral impedance signals, from which a disturbance coefficient is calculated to quantify dynamic changes related to cerebral edema bedema progression. Previous preclinical and clinical studies have demonstrated its potential for early detection of cerebral edema-related physiological deterioration [16].

Together, PF and EF provide complementary information, with PF reflecting dynamic vascular reactivity and EF representing tissue structural changes, enabling multidimensional characterization of pediatric brain injury severity.

### Model development and severity stratification

Time-series MBFM data were segmented using a 10-second sliding window and summary features (mean, variance, and temporal variability) were extracted from PF, EF, ICP, and rSO_2_. Unsupervised K-means clustering was performed to identify physiological phenotypes with the optimal cluster number determined using silhouette and Calinski–Harabasz indices [17]. A decision tree classifier was subsequently developed using cluster assignments as outcome labels to generate interpretable severity stratification rules. Model performance was evaluated by 10-fold cross-validation, and feature importance was assessed using Gini impurity reduction. All analyses were conducted in R (version 4.3.1) [18].

### Diagnostic performance of PF for predicting adverse outcomes

The primary was unfavorable neurological outcome or death during PICU hospitalization, assessed by the Glasgow Outcome Scale (GOS) at discharge. Secondary outcomes included prolonged mechanical ventilation (> 7 days) and intracranial hypertension (ICP > 20 mmHg for > 2 h). Six-month neurological outcomes were assessed by structured telephone follow-up, with favorable outcome defined as GOS ≥ 4 and unfavorable outcome as GOS < 4.

The prognostic performance of PF was evaluated using ROC curve analysis. The optimal PF cutoff was determined by the Youden index and sensitivity, specificity, positive predictive value (PPV), and negative predictive value (NPV) were calculated. The discriminative performance among PF, EF, ICP, and rSO_2_ was compared using DeLong’s test for correlated ROC curves with AUC and 95% confidence intervals reported.

### Association of multimodal parameters with clinical outcomes

The prognostic associations of multimodal parameters were evaluated using patient-level summary metrics, including mean values and dynamic ranges of PF, ICP, and rSO_2_ during 72-hour monitoring period. For rSO_2_ analysis, both absolute values and baseline-normalized changes were assessed to account for inter-individual variability. Baseline decline was defined as the difference between the first-hour mean rSO_2_ and the lowest 1-hour mean value during monitoring [19]. Multivariate logistic regression models were used to evaluate the association between PF-derived physiological states and unfavorable outcomes, adjusting for age, baseline Glasgow Coma Scale (GCS), and duration of mechanical ventilation.

**Fig. 1:**
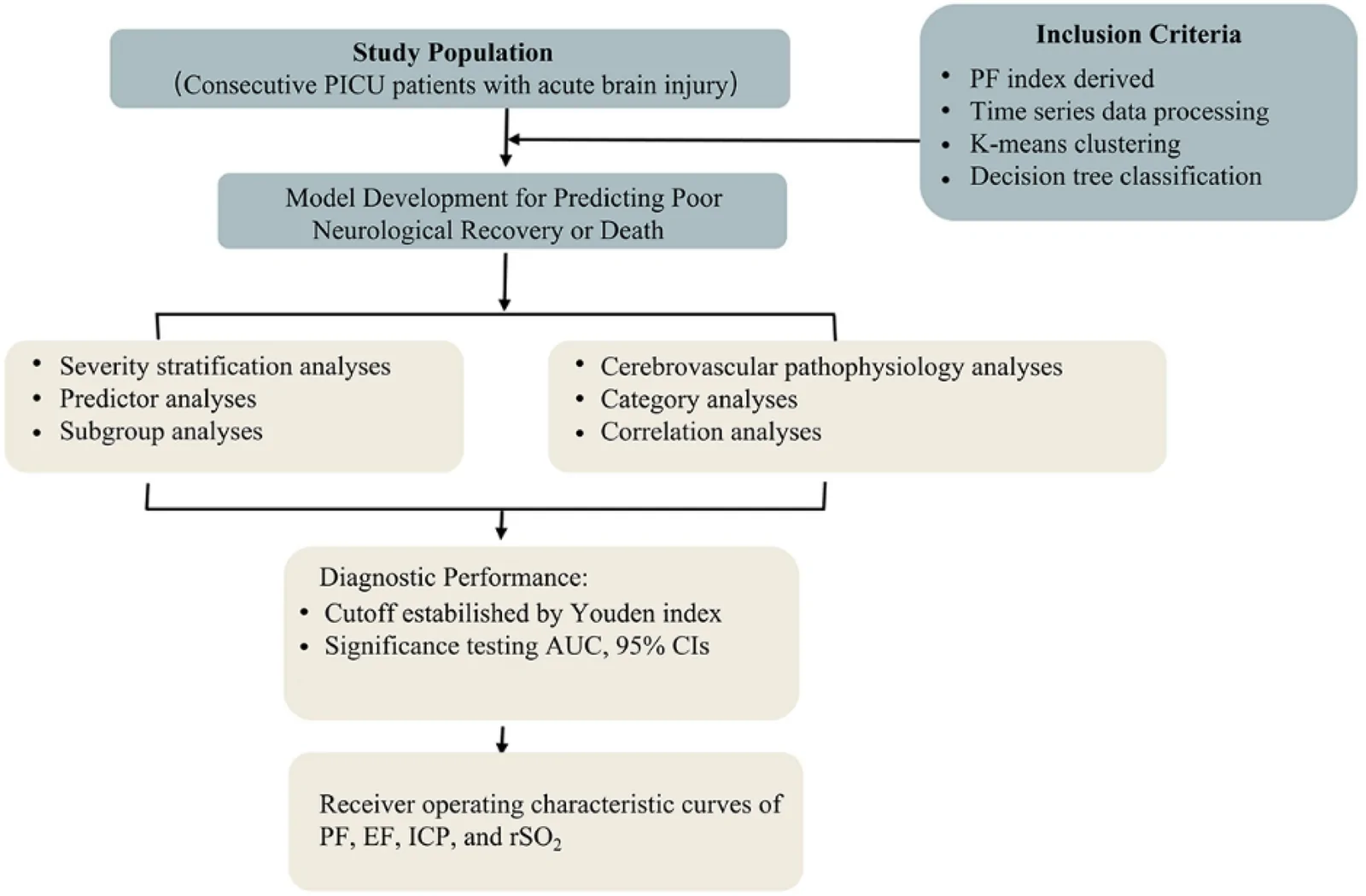
Flowchart depicting the methodological approaches and associated analyses of the study methodology.

Correlation analyses were performed to assess relationships among multimodal parameters. Etiological-stratified analyses (traumatic, hypoxic-ischemic, infectious causes) were conducted to evaluate the robustness of PF-based classification. A flowchart summarizing the overall study design, multimodal brain function monitoring procedures, data preprocessing, and severity stratification approach is presented in Figure 1.

### Neuroimaging acquisition and analysis

Neuroimaging was performed as part of routine clinical care. All patients underwent baseline non-contrast head CT on admission, while follow-up CT or MRI was obtained when clinically indicated, including neurological deterioration, failure to improve, or before ICP monitoring withdrawal.

MRI was performed using a 3.0-T scanner when patients were clinically stable and could be safely transported. For patients with ICP monitoring, MRI was performed only after ICP remained < 20 mmHg for at least 6 h with continuous physiological monitoring. Imaging findings were independently reviewed by neuroradiologists blinded to MBFM data. Representative physiological signals (PF, EF, ICP, and rSO₂) were temporally aligned with imaging time points for descriptive assessment of physiological-radiological relationships.

### Statistical analysis

Continuous variables were presented as mean ± standard deviation or median (interquartile range), as appropriate. Normality was assessed using the Shapiro–Wilk test. Between-group comparisons were performed using Student’s *t*-test or Mann-Whitney U test for continuous variables, and χ² or Fisher’s exact test for categorical variables. Correlations were assessed by Pearson or Spearman analysis.

Multivariate regression models were used to identify independent predictors of poor neurological outcomes, with model performance evaluated by calibration and discrimination analyses. ROC curves were generated to assess predictive performance, with two-sided P < 0.05 considered statistically significant.

## Results

### Patient baseline characteristics and multimodal brain function monitoring

A total of 120 pediatric patients with neurological critical illness were enrolled, comprising 70 boys and 50 girls (median age, 4.4 ± 2.6 years; range, 0.5–12.0 years). The median admission GCS score was 7.6 ± 2.1, and mean ICP was 15.2 ± 4.7 mmHg. The averaged PF, EF, and rSO_2_ were 0.32 ± 0.08, 0.27 ± 0.06, and 63.5 ± 5.8%, respectively. Baseline demographics and clinical characteristics were comparable among groups (P > 0.05) (Table 1).

**Table 1:** Baseline characteristics of pediatric patients and brain function monitoring parameters at admission ($\bar{x}$± s/ n (%)).

Parameter	Mild group (n = 42)	Moderate group (n = 48)	Severe group (n = 30)	*P* value
Sex (Male/Female)	25/17	28/20	17/13	0.96
Age groups [n (%)]				0.85
6 months-3 years	15 (35.7)	18 (37.5)	11 (36.7)	
> 3 - 6 years	19 (45.2)	21 (43.8)	13 (43.3)	
> 6 −12 years	8 (19.0)	9 (18.8)	6 (20.0)	
Age (years)	4.4 ± 2.6	4.3 ± 2.6	4.4 ± 2.7	0.94
Weight (kg)	15.5 ± 5.2	15.4 ± 5.3	15.7 ± 5.0	0.93
Admission GCS Score	8.4 ± 1.9	7.5 ± 2.0	6.1 ± 2.2	0.07
ICP (mmHg)	13.8 ± 3.9	15.5 ± 4.2	17.2 ± 5.1	0.08
PF	0.27 ± 0.05	0.34 ± 0.06	0.41 ± 0.07	<0.01
EF	0.23 ± 0.04	0.28 ± 0.06	0.34 ± 0.08	<0.01
rSO_2_(%)	66.5 ± 4.2	63.1 ± 5.0	58.4 ± 6.1	<0.01

**Fig. 2:**
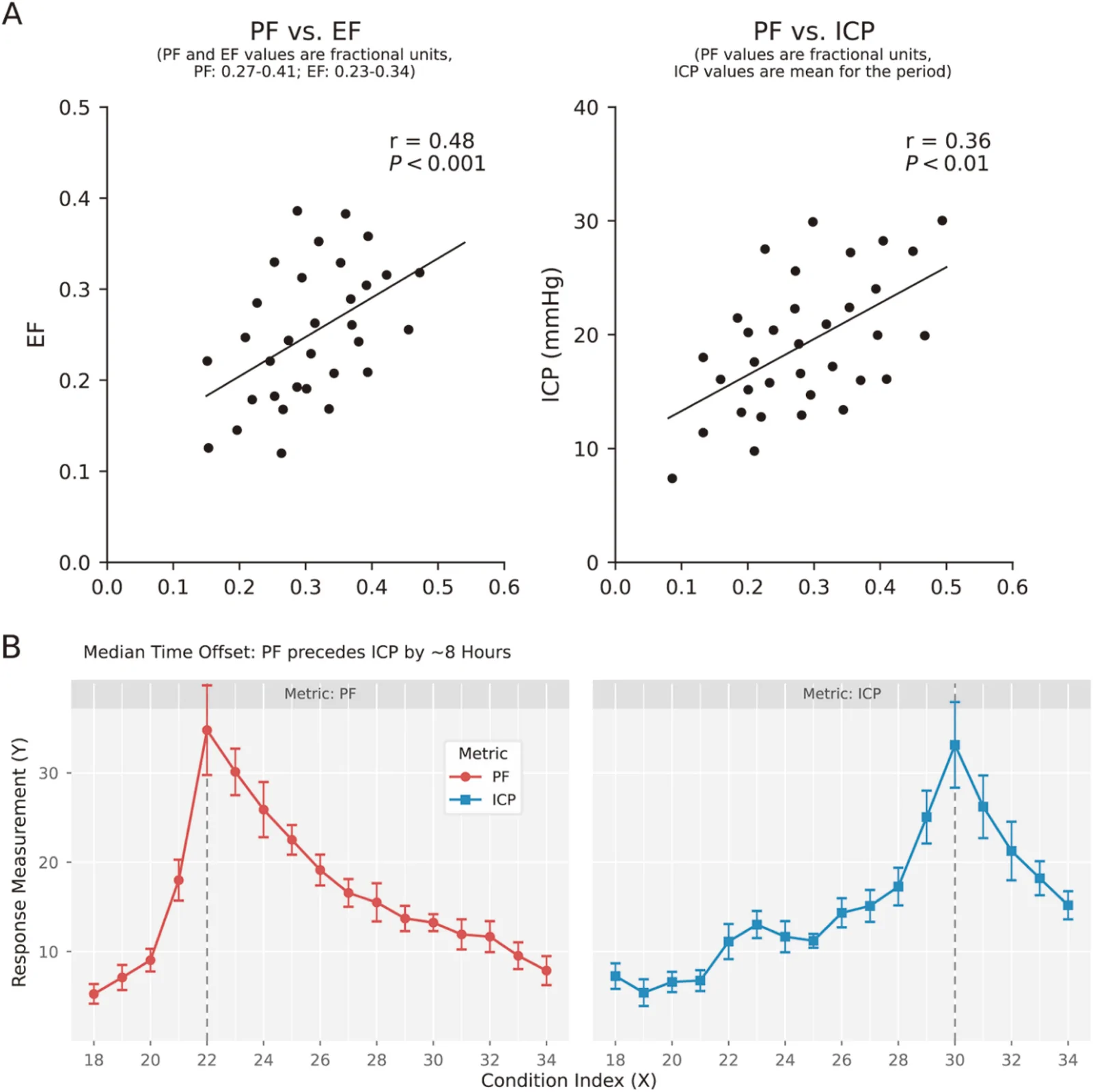
**Multimodal brain function monitoring in critically ill patients.** (A) Correlation analysis showing a moderate positive association between PF and EF (r = 0.48, P < 0.001) and a weak-to-moderate positive correlation between PF and ICP (r = 0.36, P < 0.01). (B) Temporal dynamics of intracranial pressure (ICP), perturbation factor (PF), and edema factor (EF) during the monitoring period. Peak values of ICP, PF, and EF post-admission are indicated. The ICP peak occurred at 30 h, the PF peak at 22 h, and the EF peak coincided with the ICP peak, suggesting a clear temporal relationship between cerebral edema and hemodynamic disturbances during the critical phase. Data are presented as mean ± standard deviation.

Correlation analysis demonstrated a moderate positive association between PF and EF (r = 0.48, P < 0.001) and a mild-to-moderate positive correlation between PF and ICP (r = 0.36, P < 0.01) (Figure 2A). Temporal profiling of multimodal monitoring revealed that PF reached its peak 22 h post-admission, preceding the ICP peak (30 h), while EF showed synchronized dynamics with ICP (Figure 2B). These time-dependent trajectories delineate the dynamic coupling between cerebral edema progression and hemodynamic perturbation during the critical phase.

### Data-driven stratification of multimodal brain function indices

**Fig. 3:**
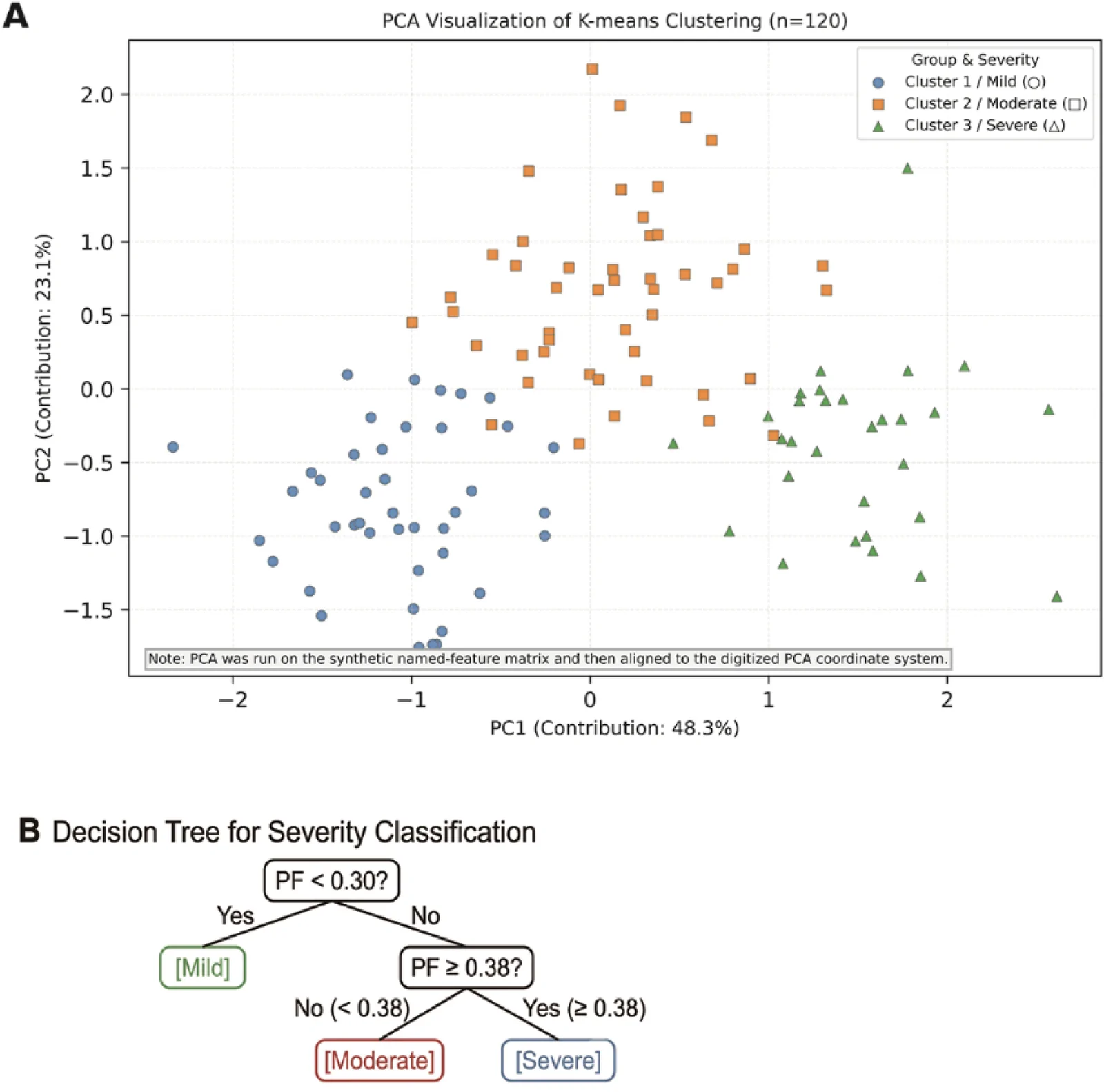
**Patient stratification based on multimodal brain function indices.** (A) K-means clustering of Z-score–standardized PF, EF, ICP, and rSO_2_ values identified three patient groups: mild (n = 42), moderate (n = 48), and severe (n = 30). Each dot represents an individual patient, with colors indicating different severity clusters. (B) Decision tree (CART) analysis identified PF as the most critical determinant for stratification, with a threshold of 0.30 separating mild from moderate cases and 0.38 separating moderate from severe cases. ICP and EF contributed secondary discriminative value, while rSO_2_ was markedly decreased in severe patients, further enhancing classification precision.

Unsupervised K-means clustering based on PF, EF, ICP, and rSO_2_ identified three distinct physiological phenotypes: mild (n = 42), moderate (n = 48), and severe (n = 30) (Figure 3A). Cluster stability was confirmed by silhouette coefficient (range = 0.41–0.63) and Calinski-Harabasz index (range = 184–376).

Decision tree analysis identified PF as the primary discriminator of disease severity, with threshold of 0.30 separating mild, moderate, and severe groups (Figure 3B). ICP and EF provided additional discrimination, while reduced rSO_2_ contributed to classification of severe phenotypes.

### Diagnostic performance of the perturbation factor

**Fig. 4:**
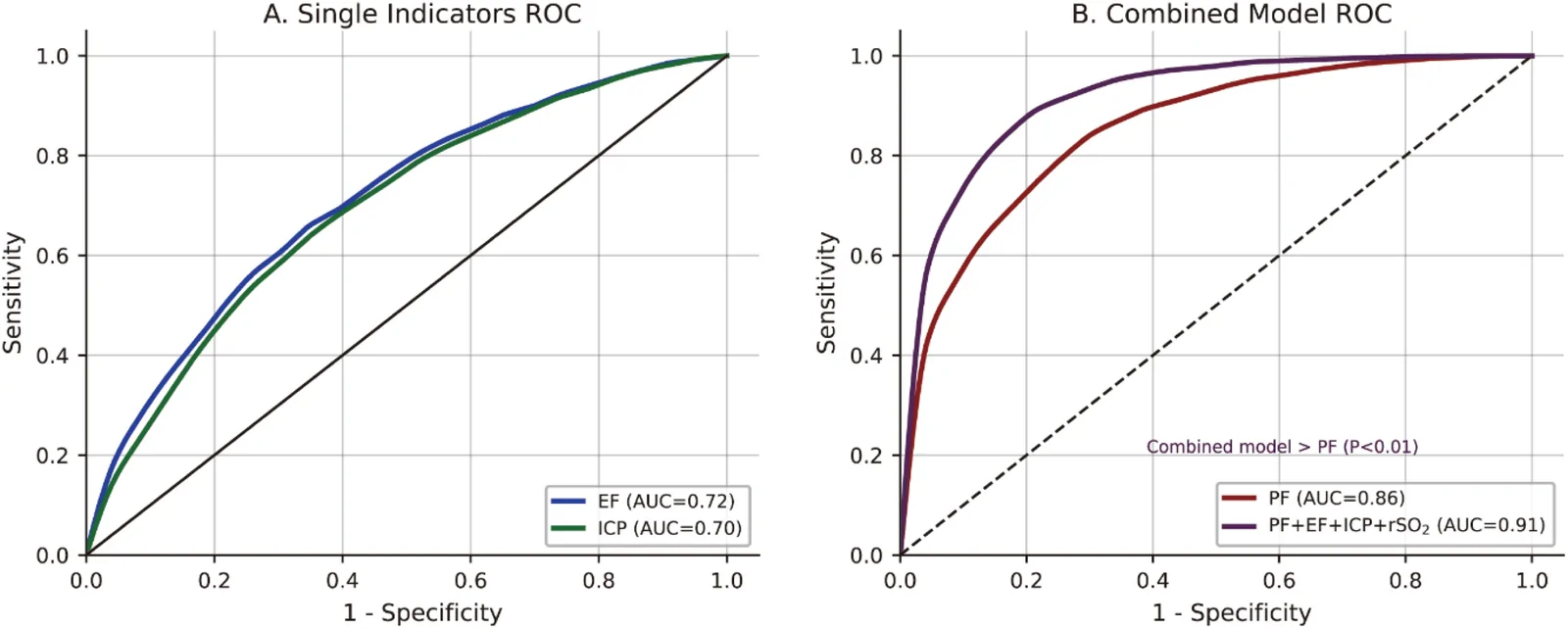
**ROC curve analysis for predicting adverse outcomes.** (A) Single-parameter ROC curves. ROC curves depicting the predictive performance of PF, EF, and ICP for adverse outcomes. The x-axis represents the false-positive rate (1 − specificity), and the y-axis represents the true-positive rate (sensitivity). Curve colors and AUC values are as follows: PF (red, AUC = 0.86, 95% CI 0.79–0.92), EF (blue, AUC = 0.72), and ICP (green, AUC = 0.70). The optimal PF cutoff (0.34) is indicated by a black dot, with a dashed line pointing to the corresponding coordinates. (B) ROC curves for the combined model (PF + EF + ICP + rSO_2_). Axes are the same as in (A). Curve colors and AUC values are: PF alone (red, AUC = 0.86) and the combined multimodal model (purple, AUC = 0.91, 95% CI 0.85–0.96).

ROC analysis showed that PF predicted unfavorable outcomes with an AUC of 0.86 (95% CI, 0.79–0.92). A PF cutoff of 0.34 yielded 85% sensitivity and 78% specificity (Figure 4A). EF and ICP showed lower predictive performance (AUC = 0.72 and 0.70, respectively; P < 0.01 vs. PF). The Integrated model combining PF, EF, ICP and rSO_2_ further improved discrimination, achieving an AUC of 0.91 (95% CI, 0.85–0.96) (Figure 4A and B; Table 2).

**Table 2:** Diagnostic performance of individual parameters and the combined model for predicting adverse outcomes.

Parameter	AUC	95% CI	Optimal cutoff	Sensitivity (%)	Specificity (%)	PPV (%)	NPV (%)	Comparison with PF (*P* value, DeLong test)
PF	0.86	0.79–0.92	0.34	85	78	73	88	—
EF	0.72	0.64–0.80	—	—	—	—	—	<0.01
ICP	0.7	0.62–0.78	—	—	—	—	—	<0.01
PF + EF + ICP + rSO₂	0.91	0.85–0.96	—	—	—	—	—	—

### Association of multimodal indices with clinical outcomes

At 6-month follow-up, 88 patients achieved favorable outcomes (GOS ≥ 4) and 32 experienced poor outcomes (GOS < 4). PF exhibited the strongest negative correlation with GOS (r = −0.59, P < 0.001), followed by EF (r = −0.41, P < 0.01) and ICP (r = −0.33, P < 0.05), whereas rSO_2_ correlated positively (r = 0.45, P < 0.01) (Table 3).

**Table 3:** Association of pediatric multimodal parameters with clinical outcomes.

Parameter	Pearson correlation with GOS (r)	*P* value	Multivariate logistic regression (Independent predictor: Yes/No)	OR	95% CI	*P* value
PF	−0.59	<0.001	Yes	4.28	2.12–8.62	<0.001
EF	−0.41	<0.01	Yes	2.31	1.12–4.76	0.023
ICP	−0.33	<0.05	No	—	—	0.081
rSO₂	0.45	<0.01	Yes	0.69	0.50–0.95	0.027

Multivariable logistic regression adjusting for age, sex, and etiology identified PF as an independent predictor of poor prognosis (OR = 4.28, 95% CI = 2.12–8.62, P < 0.001). EF (OR = 2.31, 95% CI = 1.12–4.76, P = 0.023) and rSO_2_ (OR = 0.69, 95% CI = 0.50–0.95, P = 0.027) remained significant, whereas ICP did not reach statistical significance (P = 0.081). The negative correlation between rSO_2_ and poor prognosis (OR < 1) is consistent with its positive correlation with GOS-both indicate that higher rSO_2_ is associated with better outcomes. Specifically, the OR of 0.69 means that each 1% increase in rSO_2_ is associated with a 31% reduction in the odds of poor prognosis, which aligns with the findings that higher rSO_2_ correlation with higher (favorable) GOS scores. These findings position PF as a robust independent biomarker for outcome prediction within the multimodal monitoring framework.

### Survival analysis

**Fig. 5:**
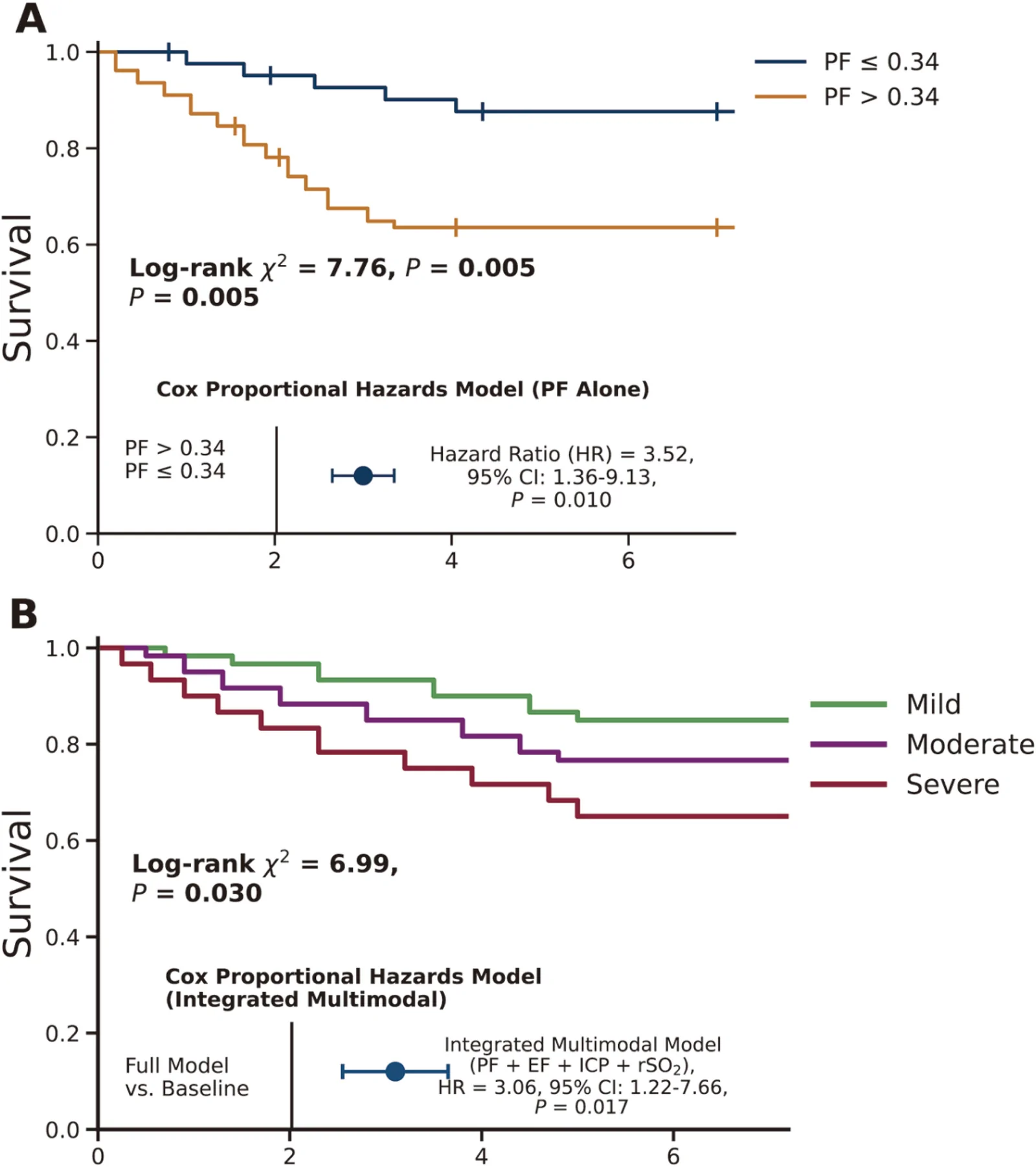
**Survival analysis based on multimodal brain function monitoring.** (A) Kaplan-Meier survival curves stratified by perturbation factor (PF). (B) Kaplan-Meier survival curves stratified by severity groups derived from multimodal indices.

Kaplan-Meier survival curves demonstrated significantly reduced 6-month survival among patients with PF > 0.34 compared to those with PF ≤ 0.34 (log-rank χ² = 7.76, P = 0.005; Figure 5A). Survival probabilities decreased progressively across severity clusters (mild > moderate > severe; log-rank χ² = 6.99, P = 0.030; Figure 5B). Cox proportional hazards modeling confirmed PF as an independent mortality risk factor (HR = 3.52, 95% CI = 1.36–9.13, P = 0.010), while the integrated multimodal model (PF + EF + ICP + rSO_2_) achieved an even higher predictive strength (HR = 3.06, 95% CI = 1.22–7.66, P = 0.017). Collectively, these survival-based analyses demonstrate that PF-centered multimodal assessment provides superior prognostic stratification compared with single-parameter approaches, as reflected by the higher hazard ratio and greater separation of survival curves across severity clusters.

### Dynamic patterns of multimodal parameters and biochemical correlates

**Fig. 6:**
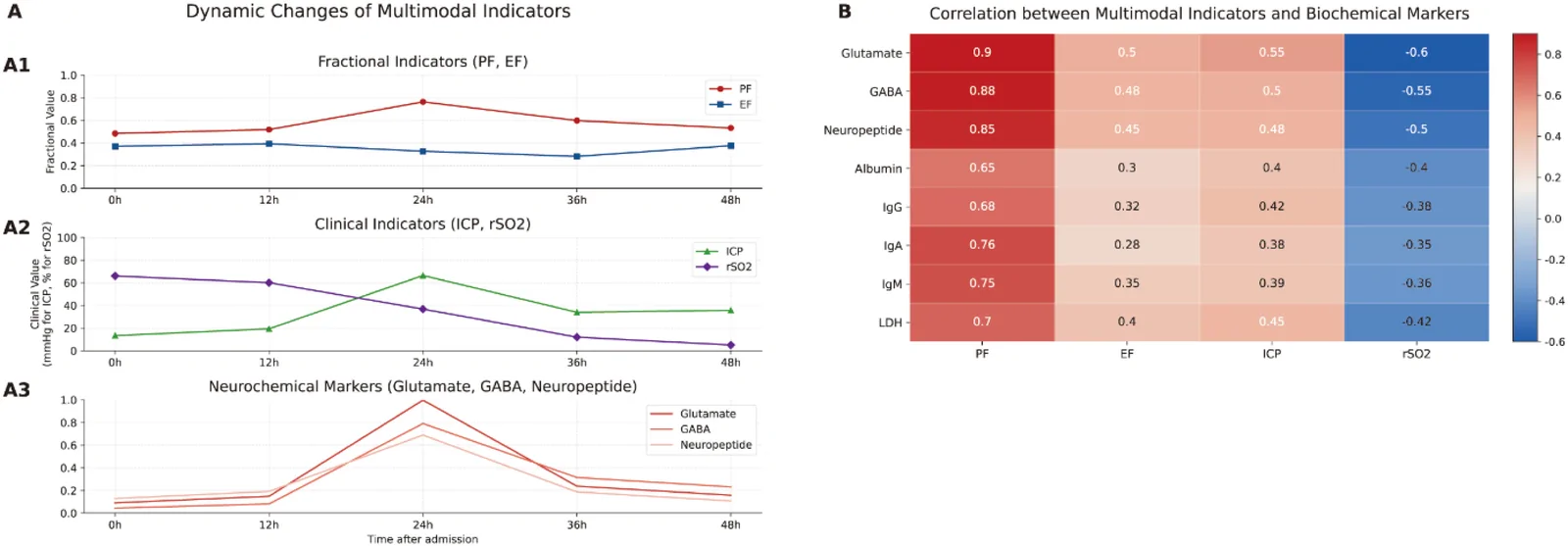
**Temporal dynamics and multimodal-biochemical correlations.** Left: Line plots showing time-dependent changes of multimodal indices (PF, EF, ICP, rSO_2_) during the monitoring period. Right: Heatmap illustrating correlations between PF, EF, ICP, rSO_2_ and serum biomarkers, including glutamate, GABA, neuropeptides, and immune-metabolic indicators.

Longitudinal analysis revealed synchronized PF, EF, and ICP fluctuations within the first 24–36 h post-admission, coinciding with a marked decline in rSO_2_ among critically ill patients. Plasma glutamate, GABA, and neuropeptide dynamics mirrored PF trajectories. Heatmap correlation analysis (Figure 6) linked PF with systemic markers including albumin, immunoglobulins (IgG, IgA, IgM), and LDH. These multimodal-biochemical correlations suggest that real-time perturbation metrics reflect both intracranial dynamics and systemic neuro-metabolic and inflammatory changes in critical states.

### Neuroimaging characteristics

**Fig. 7:**
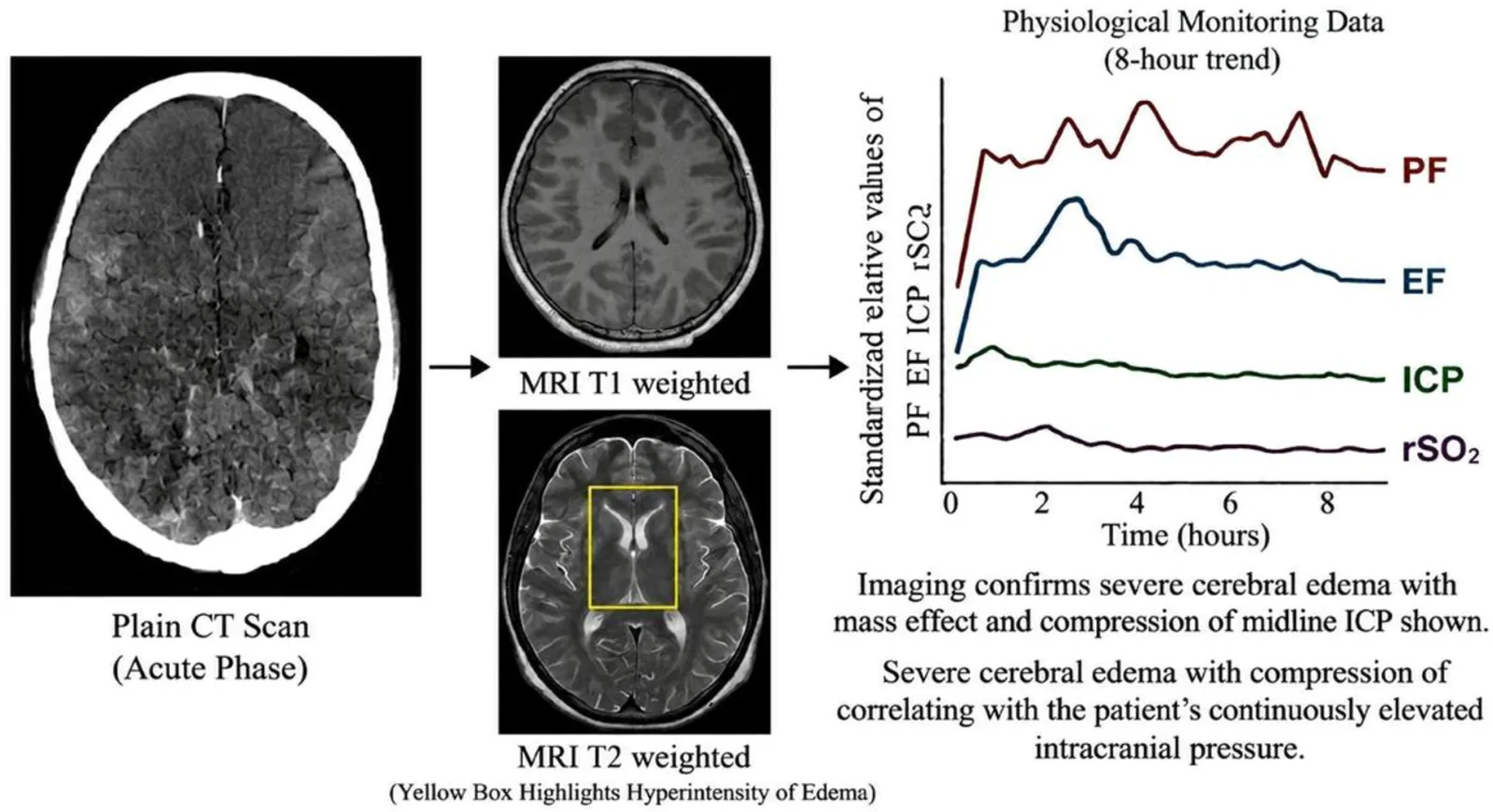
**Representative multimodal monitoring profile of a patient with severe cerebral edema and intracranial hypertension.** Representative CT and MRI images showing cerebral edema, including acute-phase plain CT, T1-weighted MRI, and T2-weighted MRI with the highlighted edema region (yellow box). Continuous 8-hour physiological monitoring profiles of intracranial pressure (ICP), regional cerebral oxygen saturation (rSO_2_), edema factor (EF), and perturbation factor (PF) are presented.

Neuroimaging corroborated these physiological findings. In severe cases, CT revealed diffuse edema, midline shift, and raised ICP, while MRI T2-weighted and FLAIR sequences identified focal ischemia and edema expansion. Representative multimodal traces (PF, EF, ICP, and rSO_2_) aligned with the timing of neuroimaging acquisition illustrated synchronous physiological alterations corresponding to radiologically confirmed deterioration-for example, rising PF and EF preceding CT-confirmed edema progression, and declining rSO_2_ corresponding to MRI-detected ischemic changes (Figure 7), underscoring the complementary diagnostic value of continuous multimodal monitoring in identifying high-risk neurological deterioration.

## Discussion

Based on the integrated multimodal monitoring of PF, EF, ICP, and rSO_2_, we identified three distinct physiological phenotypes in a prospective cohort of pediatric neurocritical patients using unsupervised clustering and decision-tree analysis. These phenotypes demonstrated clear separation in cerebral hemodynamics, oxygenation status, and functional reactivity, and were consistently associated with clinical outcomes across etiological subgroups.

Cluster formation was driven exclusively by physiological parameters measured within the first 48 h after admission, excluding outcome information from model training. This design enabled unbiased assessment of how early multimodal profiles predict subsequent clinical trajectories. With respect to clinically relevant endpoints such as unfavorable outcome or mortality, clusters exhibited distinct prognostic patterns ranging from mild (Cluster I) to moderate (Cluster II) to severe (Cluster III) phenotypes [20,21]. A key findings of this study is that early multimodal physiological patterns provide meaningful prognostic stratification in independent of diagnostic category. Cluster III, characterized by elevated PF, increased ICP and EF, and persistently reduced rSO_2_, was strongly associated with unfavorable neurological outcomes and lower survival. In contrast, Cluster I exhibited stable PF, preserved oxygenation, and near-normal ICP dynamics, corresponding to favorable recovery. Cluster II represented an intermediate state with transient dysregulation and partial compensatory recovery. Importantly, PF changes preceded ICP elevation by approximately 6–12 h, suggesting that functional neurovascular instability occurs before overt intracranial hypertension. Encouragingly, the prognostic performance and cluster structure of our multimodal framework remained stable across age strata, suggesting that the physiological phenotypes identified are not merely artifacts of developmental heterogeneity. Nevertheless, the relatively small sample size within each age subgroup precluded definitive establishment of age-specific normative thresholds. These findings extend prior work in pediatric neurocritical care, which has largely focused on single-modality monitoring such as ICP or cerebral oxygenation threshold [22,23]. Such approaches implicitly assume linear and independent relationships among physiological variables [24], neglecting the biological reality that cerebrovascular autoregulation, neural electrical activity, and metabolic coupling are interdependent and dynamically evolving processes [13,25]. By contrast, our PF metric quantifies neurovascular coupling perturbations and integrates multimodal signals. Unlike the “one-parameter-one-decision” paradigm, PF-based clustering treats these signals interactively, revealing relationships among functional, hemodynamic, and metabolic alterations that conventional frameworks miss.

Recent adult and neonatal studies have suggested that multimodal integration improves detection of secondary brain injury, but most remain descriptive and lack unified physiological phenotyping frameworks [26,27]. Our results demonstrate that combining PF with ICP, EF, and rSO_2_ enables data-driven identification of clinically meaningful brain states with improved predictive performance (AUC 0.91), outperforming single-parameter models. Notably, the additive value of rSO_2_ in our model should be interpreted with caution, as absolute rSO_2_ values are known to have limited correlation with invasive brain tissue oxygen tension (PbO_2_) in some studies. Nevertheless, as a non-invasive, continuous trend-monitoring modality, rSO_2_ provided complementary information on oxygenation trajectory that enhanced cluster separation when combined with other physiological indices.

From a clinical perspective, these findings support a shift from threshold-based monitoring toward phenotype-guide risk stratification [28]. Early identification of high-risk physiological profiles (e.g., high PF cluster) may allow earlier optimization of cerebral perfusion and prevention of secondary injury, whereas stable profiles may benefit from less aggressive intervention [29]. The ability of PF to anticipate ICP elevation suggests potential utility as an early warning biomarker of impending decompensation, though prospective interventional studies are needed to establish whether PF-guided management improves outcomes.

This study has several limitations. First, it is a single-center observational study, which may limit generalizability. Second, although clustering was performed without outcome input, residual confounding from heterogeneous etiologies (trauma, infection, hypoxia) may influence physiological trajectories. Third, we lacked independent gold-standard measurements of cerebral blood flow and metabolism, such as transcranial Doppler or metabolic imaging, which limit direct physiological validation of PF and EF. Fourth, while PF showed strong predictive performance, inter-device and inter-center standardization remains necessary before clinical implementation. Fifth, although we performed age-stratified analyses to address developmental heterogeneity, the sample size within each age stratum was limited, and the optimal age-binning scheme (6 months-3 years, > 3–6 years, > 6–12 years) requires external validation. Future multicenter studies with larger samples are needed to establish formal age-adjusted normative values for PF, EF, and their integrated phenotypes. Sixth, the lack of racial and ethnic diversity in our single-center Chinese cohort limits the generalizability of our findings to other populations. While the physiological mechanisms assessed are unlikely to be population-specific, the applicability of specific threshold values (e.g., PF cutoff 0.34) and phenotype definitions to ethnically diverse groups requires prospective validation. Finally, the observational design of this study precludes causal inference regarding whether PF-guided interventions improve outcomes-a critical question that must be addressed in future interventional trials.

In summary, multimodal physiological phenotyping using PF, EF, ICP, and rSO_2_ provides clinically meaningful stratification of pediatric neurocritical states and improves early risk prediction compared with conventional single-parameter monitoring. These findings support further validation of PF-guided multimodal monitoring as a potential tool for precision neurocritical care.

## Authors’ contributions

All the authors conceived the articles. Yanmei Wang, Haili Wang and Minglei Li collected the data and analyzed the information. Yanmei Wang and Haili Wang wrote the article. Xianli Shao and Huafeng Zhao collected the data and reviewed the article.

## Funding

Shandong Province Medical and Health Science and Technology Project (202306011373).

## Data availability

The data generated by this research can be obtained from the authors.

## Conflicts of interest

The authors declare no conflicts of interest.
